# Metatranscriptomic profiles of Eastern subterranean termites, *Reticulitermes flavipes* (Kollar) fed on second generation feedstocks

**DOI:** 10.1186/s12864-015-1502-8

**Published:** 2015-04-22

**Authors:** Swapna Priya Rajarapu, Jacob T Shreve, Ketaki P Bhide, Jyothi Thimmapuram, Michael E Scharf

**Affiliations:** Department of Entomology, Purdue University, West Lafayette, 47907-2089 Indiana; Bioinformatics Core, Purdue University, West Lafayette, 47907-2089 Indiana

**Keywords:** Termite, Corn stover, Soybean residue, Lignocellulase, RNA-seq, Protists

## Abstract

**Background:**

Second generation lignocellulosic feedstocks are being considered as an alternative to first generation biofuels that are derived from grain starches and sugars. However, the current pre-treatment methods for second generation biofuel production are inefficient and expensive due to the recalcitrant nature of lignocellulose. In this study, we used the lower termite *Reticulitermes flavipes* (Kollar), as a model to identify potential pretreatment genes/enzymes specifically adapted for use against agricultural feedstocks.

**Results:**

Metatranscriptomic profiling was performed on worker termite guts after feeding on corn stover (CS), soybean residue (SR), or 98% pure cellulose (paper) to identify (i) microbial community, (ii) pathway level and (iii) gene-level responses. Microbial community profiles after CS and SR feeding were different from the paper feeding profile, and protist symbiont abundance decreased significantly in termites feeding on SR and CS relative to paper. Functional profiles after CS feeding were similar to paper and SR; whereas paper and SR showed different profiles. Amino acid and carbohydrate metabolism pathways were downregulated in termites feeding on SR relative to paper and CS. Gene expression analyses showed more significant down regulation of genes after SR feeding relative to paper and CS. Stereotypical lignocellulase genes/enzymes were not differentially expressed, but rather were among the most abundant/constitutively-expressed genes.

**Conclusions:**

These results suggest that the effect of CS and SR feeding on termite gut lignocellulase composition is minimal and thus, the most abundantly expressed enzymes appear to encode the best candidate catalysts for use in saccharification of these and related second-generation feedstocks. Further, based on these findings we hypothesize that the most abundantly expressed lignocellulases, rather than those that are differentially expressed have the best potential as pretreatment enzymes for CS and SR feedstocks.

**Electronic supplementary material:**

The online version of this article (doi:10.1186/s12864-015-1502-8) contains supplementary material, which is available to authorized users.

## Background

Second generation biofuels have gained interest in the field of biofuel production due to their several advantages over the first generation biofuels. Second generation biofuels are produced from various kinds of plant biomass including agricultural/forest by-products, organic wastes and other dedicated feedstocks [1]. Lignocellulose, a complex of lignin, cellulose and hemicellulose, is the major component of plant biomass and second generation feedstocks. Breakdown of the lignocellulose matrix is required for the release of free sugars for biofuel production, and is performed in the three major steps of pretreatment, hydrolysis and fermentation. Pretreatment is the limiting step in biofuel production and involves the disintegration of lignocellulose polymers to increase the availability of cellulose and hemicellulose for downstream biochemical processes [2]. There are several physical and chemical pretreatment methods available (e.g., steam, liquid hot water, acid treatment) for lignocellulosic biomass which have been extensively reviewed [2-5]. However, the current pretreatment methods have disadvantages wherein the major concern is the inhibition of cellulolytic enzymes by lignin, mono-lignols and other phenolic metabolites in downstream processes [6,7]. To date, one of the most significant challenges to biofuel production is the development of economically efficient pretreatment processes.

Termites are efficient lignocellulose decomposers with the unique ability to utilize most of the cellulose from their diet [8-11]. The lower termite, *Reticulitermes flavipes* (Kollar), is used as a model in this study to investigate potential mechanisms of lignocellulose feedstock digestion. *R. flavipes,* along with its microbial symbionts, produces a bouquet of enzymes that participate in lignocellulose degradation [12,13]. Cellulases, hemicellulases and candidate ligninases were previously identified in *R. flavipes* [13-15]. A recombinant enzyme cocktail of *R. flavipes* enzymes was shown to result in the release of free sugars from wood substrate *in vitro* [16]. This evidence supports the further investigation and application of *R. flavipes* digestion mechanism(s) in biofuel production from lignocellulose biomasses.

In this study we evaluated changes in the metatranscriptomic profiles of the *R. flavipes* gut microenvironment in response to feeding on two second generation feedstocks (corn stover [CS] and soybean hull residue [SR]). Our hypothesis was that genes encoding key lignocellulose processing enzymes would be differentially expressed among these treatments. To test this hypothesis, we studied the gut metatranscriptomes to identify potential lignocellulase genes of *R. flavipes* workers fed on different feedstocks via the Illumina Hi-Seq platform. Our findings show a clear ability of *R. flavipes* to feed and survive on two very different plant feedstocks (CS and SR); however, transcripts encoding wood-associated lignocellulases identified through previous work were not affected by the diets. Along with these results our Illumina deep sequencing results significantly contribute to the molecular resources available for *R. flavipes*, and also shed light on novel feedstock-associated genes from *R. flavipes*.

## Methods

### Feedstock composition analysis

Lignin content in the feedstocks was estimated using the acetyl bromide method of Fukushima and Kereley [17,18]. Briefly, cell walls of the feedstocks were extracted using the following method. Finely milled feedstocks (60 mm particle size) were incubated in sodium phosphate buffer (0.1 M, pH 7) for 30 minutes at 50°C followed by centrifugation at 3000 g for 10 min. Supernatant was discarded and the pellet was washed with 70% ethanol at 70°C for 5 to 6 times. One volume of acetone was added and centrifuged at 3000 g for 10 minutes. Supernatant was discarded and pellet was dried in oven at 45 ± 5°C for overnight. Approximately 5 mg of dried cell wall was dissolved in 2.5 ml of 25% acetyl bromide v/v in glacial acetic acid at 70°C for 30 min in a glass tube closed with a Teflon lined cap. Acetyl bromide with dissolved cell wall was then transferred to a 50 ml volumetric flask containing 12 ml glacial acetic acid and 2.5 ml of 2 N sodium hydroxide followed by the addition of 0.5 ml of 7.5 M hydroxylamine hydrochloride. The final volume was made to 50 ml and absorbance was measured at 280 nm in a spectrophotometer (BioRad SmartSpec™ 3000). Two feedstocks including CS and SR were run in triplicates; blanks were included to correct for background absorbance [17,18].

Carbohydrate analysis was performed following the National Renewable Energy Lab protocol with slight modifications [19]. Briefly, 300 mg of feedstock including CS and SR was hydrolyzed in 3 ml of concentrated sulfuric acid in pressure tubes at 30°C for 1 hr with occasional stirring. Hydrolysis was stopped by adding 84 ml of water and further hydrolyzed at 121°C in an autoclave for 1 hour. The resultant liquid was aliquoted in 1 ml glass vials for quantification of carbohydrates using a Waters e2695 separation module HPLC system at the Analytical chemistry center, Laboratory of Renewable Resources Engineering, Purdue University.

### Bioassays, gut removal and RNA isolation

Feeding assays were performed using *R. flavipes* workers collected from West Lafayette, Indiana (single colony) and Florida (2 colonies) which served as three independent biological replicates. Collected termites were maintained under laboratory conditions (22°C with 70% relative humidity and a 0:24 light:dark photoperiod) on wood shims and paper towels before the feeding assays. Diets used in this study were Whatman no.1 filter paper (Maidstone, UK), CS (Specialty 3557) and SR (Williams 82). The CS was donated by Dr. Nathan Mosier of Purdue University Agricultural and Biological Engineering department and SR was donated by Dr. Karen Hudson of Purdue University USDA-Agronomy department. Diets were ground into a fine powder using a DCG-20 coffee grinder (Cuisinart; Stamford, CT) and made into “cookies” using nanopure water. Cookies were then dried at 50 ± 5°C for 48 hr and weighed before feeding. Feeding assays were performed in 35 mm Petri dishes with 30 termites per diet treatment. Three independent replicates were performed per treatment. After 7 days, the weight of termites and any remaining diet were recorded after the assay period to account for diet consumption. Whole termite guts including symbionts were then removed and pooled in RNA isolation buffer, and total RNA extracted with the SV Total RNA Isolation System (Promega; Madison, WI). Total extracted RNA (>10 μg) was assessed for quality using a Nanodrop spectrophotometer (Thermo Scientific) and submitted to the Purdue University Genomics Core Facility (PGCF) for cDNA library synthesis and sequencing.

### Illumina sequencing and bioinformatics analysis

Two microliters of the total RNA was further analyzed for quality using a Bioanalyzer (Agilent Inc.). One microgram each of total RNA was enriched for mRNA using polyT hybridization and cDNA libraries were bar-coded for all the 9 replicate samples by the PGCF using TruSeq™ RNA sample preparation kit (Illumina). The bar-coded libraries were paired end sequenced with a read length of 150 per read using the Illumina Hiseq2500 platform. Adapters were removed using Trimmomatic and pre-processed for downstream analysis. Low quality bases (Phred33 score of < 30) were trimmed using FASTX toolkit (v 0.0.13) [20] after accessing the quality using FastQC (v 0.10.0) [21]. Annotation of raw reads was performed in a hierarchy for maximum annotation. The first annotation method used was MetaCV (v 2.2.9) analysis using a database of protist and termite protein sequences from NCBI [22]. Previous cDNA sequencing of *R. flavipes* gut tissue by Tartar et al. [23] has identified a significant number of bacterial sequences in a symbiont library and thus the remaining reads were processed with MetaCV using a second database of all bacterial protein sequences extracted from NCBI. The non-annotated reads were joined using FastqJoin (v 1.1.2-537) and in-house perl scripts. The entire set of joined Fastq reads was further analyzed using BLAST against termite and protist database (v 2.2.28) to further classify the joined reads that failed to annotate during each round of MetaCV [24]. The remaining joined reads that could not be annotated from BLAST were used as input for RAPSearch2 (v 2.12) [25]. Each annotation step resulted in GIs (GenInfo Identifiers), which were used to extract genus and species names using the BLAST tool blastdbcmd. An abundance matrix containing counts was generated for all unique genera. Differential analysis of genera abundance for pairwise comparisons of all 3 treatments were conducted using R (version 3.0.3) and the edgeR (v 3.4.2) package with default parameters [26,27].

In a separate pipeline, total reads from all the libraries were joined via FastqJoin (v 1.1.2-537) and were uploaded into MG-RAST (Metagenomics – Rapid Annotation using Subsystem Technology [28]) through a private repository. The data were compared to M5NR database using a maximum e-value of 1e-5, a minimum identity of 60%, and a minimum alignment length of 15 measured in amino acids for protein and base pairs for RNA databases. The functionally annotated reads obtained from MG-RAST were used to generate a pathway and KO (KEGG orthology) term count matrix to evaluate differential functional expression using edgeR (v. 3.4.2). A Principal Co-ordinate Analysis (PCoA) was performed for genera and functional abundance profiles using Bray-Curtis distance measures for genera abundance and Euclidean distance measures for functional abundance using MG-RAST tool. Bacterial abundances were analyzed using best hit classification method in MG-RAST. Analyses of significant differences between bacterial phyla were carried out using two non-parametric tests ANOSIM [29] and Adonis [30]. Bacterial abundance values were converted to a Bray-Curtis distance matrix using the ‘vegdist’ function from vegan package (Version 2.0.10) (R version 3.1.1). This distance matrix was used as an input for ANOSIM (Analysis of Similarity) and Adonis test. Proc GLM (General Linear Model) Analysis of variance (ANOVA) with “feedstocks” as the main effect was used to analyze the differences between bacterial abundance among the diets (Paper, CS and SR) in SAS (version 9.2). Data were transformed when required to follow normal distribution.

### Protist counts

A post-hoc feeding assay was set up with 10 termites per treatment per colony as described earlier, using five different lab-maintained termite colonies which were in the lab for a similar time period as the sequenced colonies. Protists were counted from the hindguts of termites using a Sedgewick rafter cell counter. Hindguts of termites were collected in 1X phosphate buffered saline (100 μl/gut). The resultant stock solution was diluted appropriately to 1 ml to achieve a countable dilution of cells per square of the cell counter. Cells were counted using a phase contrast microscope under the 20X objective. The final calculation for cell count in the stock solution was carried out using the following formula: cells/ml = (dilution factor) (cells counted) (calibration constant)/number of squares containing cells counted [31]. Mean counts were compared statistically using non-parametric Kruskal-Wallis tests followed by a Tukey’s test for pairwise comparisons (Sigma plot v11.0).

### RT-qPCR validations

Differentially expressed genes along with the abundantly expressed putative lignocellulases including the Cell −1 (endoglucanase), GHF-7 (Cellobiohydrolase) were validated using RT-qPCR. Primers were designed using Primer3 [32] (Additional file 1: Table S1). Feeding bioassays were repeated with the same experimental design as mentioned earlier and three different *R. flavipes* colonies collected from West Lafayette, Indiana were used, which were also assessed for protist counts. Validations were performed on the sequenced samples and also the repeated experimental replicates (i.e., new biological samples). Total RNA isolated from each experimental replicate was used to synthesize first strand cDNA using the iScript cDNA synthesis kit (BioRad). RT-qPCR reactions were performed in a 10 μl reaction mixture containing 1 μl of cDNA template, 0.5 μl of each forward and reverse primers (10 μM), 5 μl SYBR green mastermix (2X, BioRad) and 3 μl of nuclease free water. All the primers designed for validation were tested a priori for specificity using PCR and for efficiency using standard curves of diluted template *R. flavipes* specific translation elongation factor delta (*TEF-δ*) was tested for stability across the treatments and used as the reference gene in this study. Thermocycler conditions for RT-qPCR were: 95°C for 3 min, 40 cycles of 95°C for 30 sec, 60°C for 30 sec, and 72°C for 30 sec. Relative fold changes were calculated using delta Ct method [33]. Fold changes from RNA-Seq and RT-qPCR are compared using a paired *T*-test (Sigma plot v11.0). A Spearmann correlation was performed for the fold changes of RNA-seq and RT-qPCR. Correlation was also tested for RT-qPCR fold changes from Illumina sequenced samples and experimentally replicated samples (i.e., new biological samples).

## Results

### Feeding observations

**Figure 1:**
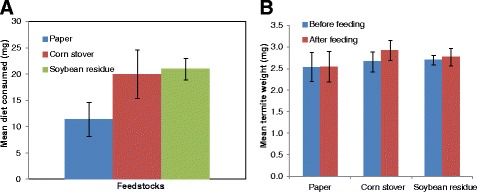
Feeding bioassay results for the same termites used in metatranscriptome analyses. Summary of 7-day feeding assays results for groups of *Reticulitermes flavipes* workers fed diets of paper, corn stover or soybean residue. **(A)** Dry weight consumption of diets over entire assays. **(B)** Average weights of termites before and after feeding assays. Graph source: Amit Sethi.

Worker termites feeding on diets of paper, corn stover (CS) and soybean residue (SR) had 100% survivorship across all replicates after 7 days of feeding bioassays. These were the same termites used for Illumina sequencing as reported below. Interestingly, termites feeding on CS and SR consumed two times more on a per weight basis relative to termites feeding on paper (Figure 1). This observation is supported by the compositional analysis of feedstocks wherein CS and SR have relatively lesser carbohydrates (glucose and xylose) and more lignin content than paper (Additional file 1: Table S2).

### Bioinformatics analyses

Illumina sequencing resulted in approximately 800 million reads (400 million paired-end reads) in total from all treatments and replicates (Additional file 1: Table S3). An informatics pipeline involving several steps and analysis platforms was used for analysis (Additional file 2: Figure S1). Out of the total reads obtained from all the treatments, 98% of raw reads were retained after the removal of low scored reads (phred33 < 30). MetaCV searches of total reads against termite and protist databases assigned taxa identities to 17% of all reads with an e-value of 10^−3^. Annotation with MetaCV using a bacterial database annotated 2% of the total reads. Further, joining of overlapping reads that were initially non-annotated made it possible to annotate an additional 1-2% of the total reads. Specifically, BLAST searches of these joined reads against termite and protist EST databases annotated 38% of these reads with an e-value of 10^−3^. A RAPSearch2 identified 7% of the total joined reads with an e-value of 10^−3^ against a non-redundant database (Additional file 1: Table S4A).

Analysis of joined reads obtained per treatment in MG-RAST removed an average of 46% of the joined reads due to quality control screening across the treatments. Another 46% of reads per treatment library that passed quality control were putatively annotated as containing ORFs. Nine percent of the predicted ORFs per library had homology to proteins and were assigned putative protein IDs. The final step in the MG-RAST pipeline assigned 5% of total reads on average per treatment to pathways. In total, approximately 2.5 M reads per replicate per feedstock treatment were functionally annotated (Additional file 1: Table S4B). Rarefaction curves show the intensive sampling of the nine metatranscriptome libraries (Additional file 2: Figure S2). Also, a majority of the annotated sequences across the treatment libraries had similarity to phylum Arthropoda and other eukaryotes (Additional file 2: Figure S3).

**Figure 2:**
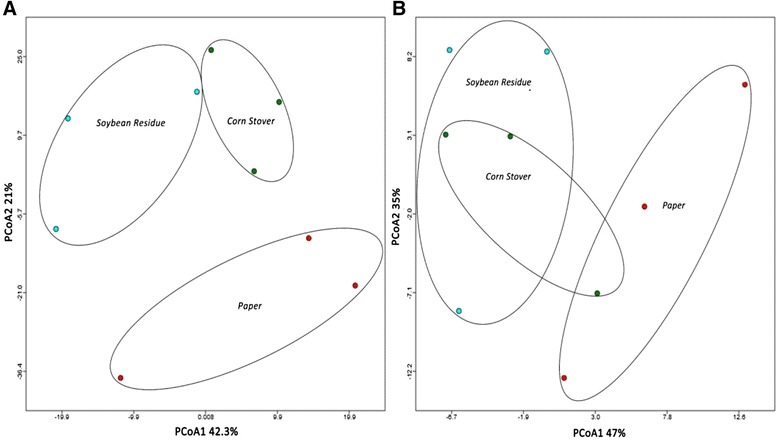
Principal Coordinate Analysis of reads from the guts of *Reticulitermes flavipes* workers fed for 7-days on paper, Corn Stover or Soybean Residue. Reads were compared to **(A)** Subsystems for genera abundance or **(B)** KO for functional abundance using a maximum e-value of 1e-5 and a minimum identity of 60%. The data were normalized to values between 0 and 1. Bay-Curtis distance measures were used for genera abundance and Euclidean distance measures used for functional abundance.

PCoA of genera abundance and functional profiles showed interesting differences among treatments (Figure 2A, B). Genera profiles of paper-fed worker guts varied from the profiles of worker guts feeding on CS and SR. Here, the first two PCoA axes explained 63.3% of variation, PCoA1 (42.3%) and PCoA2 (21%) (Figure 2A). The functional profile of CS was similar to both paper and SR; whereas, paper and SR had different functional profiles (Figure 2B). The first two PCoA axes explained 82% of variation at the functional level, PCoA1 (47%) and PCoA2 (35%). These PCoA results verify that differential metatranscriptome impacts among the three feeding treatments did indeed occur.

### Community and functional profiles

#### Genera

**Figure 3:**
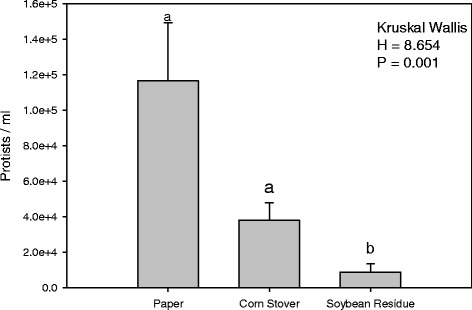
Protist counts after seven day bioassays. Protist abundance in the guts of worker termites (*Reticulitermes flavipes*) fed on diets of paper, Corn Stover or Soybean Residue for 7 days, as determined through a replicated post-hoc experiment. Mean counts were compared statistically using non-parametric Kruskal-Wallis tests and Tukey’s test for pairwise comparison. Bars with the same letters are not significantly different (*p*<0.05).

The annotated reads were grouped into 1,124 total genera across all treatments. Among these, 17 genera were differentially abundant at FDR < 0.05 between the guts of paper, CS and SR-fed termites (Table 1); of these, 13 were protists belonging to the Phylum Parabasalia, 2 were bacteria belonging to the Phyla Firmicutes and Bacteroidetes, and 2 were algae belonging to the Phylum Euglenozoa. Between CS and SR treatments, the SR-fed gut microbial community was affected the most. In the SR treatment, there was a significant reduction in the number of protist groups found in the gut, whereas CS-fed termites exhibited reductions in only one protist group. Specifically, Sarcomastigophora (protist) and Ochrophyta (algae) group members were present in significantly higher numbers in the SR treatment relative to CS. The substantial reduction in protist numbers in the guts of worker termites feeding on SR was further corroborated by independent post hoc tests using new biological samples (Kruskal-Wallis, *p* = 0.001) (Figure 3). There also was a significant effect of diet (ANOSIM, R = 0.53, *p* = 0.002; Adonis, R^2^_=_ 0.72, *p =* 0.005) on the bacterial groups sequenced in the metatranscriptome data. (Additional file 1: Table S5 and Additional file 2: Figure S4).

**Table 1:** **Community level differentials**

**Genera**	**Phylum**	**CS/Paper**	**SR/Paper**	**SR/CS**
***Protist***				
Devescovina	Parabasalia		0.30	
Eucomonympha	Parabasalia		0.10	0.08
Hoplonympha	Parabasalia		0.11	
Joenina	Parabasalia			0.39
Leishmania	Sarcomastigophora			**2.43**
Mastigamoeba	Archamoeba	0.37	0.31	
Monocercomonas	Parabasalia		0.28	0.45
Pseudotrichonympha	Parabasalia		0.11	0.24
Staurojoenina	Parabasalia		0.18	0.18
Stephanonympha	Parabasalia		0.26	
Teranympha	Parabasilia		0.11	0.09
Trichonympha	Parabasalia		0.13	0.18
uncultured protist Unknown			0.13	0.18
***Bacteria***				
Oenococcus	Firmicutes		0.42	
Ornithobacterium	Bacteroidetes			0.53
***Algae***				
Bodo	Euglenozoa	0.40		
Chlorellidium	Ochrophyta			**4.31**

Differentially abundant genera in the guts of worker termites (*Reticulitermes flavipes*) feeding on Paper, Corn Stover (CS), or Soybean Residue (SR) at FDR p < 0.05. Values greater or less than 1 are higher in the numerator or denominator of each respective comparison.

#### Function

Count matrices generated for pathway-level comparisons consisted of 353 pathways and a matrix of KO terms comprised of 4,399 KO terms generated for gene-level comparisons. An edgeR-based differential expression analysis showed 20 differentially expressed pathways in total among paper, CS and SR-fed termite guts at FDR (False Discovery Rate) < 0.05 (Table 2). False Discovery Rate is a multiple testing hypothesis developed to control false positives in the differential expression results. Termite guts fed on SR-diets were the most affected at the pathway level. Among the differentially expressed pathways, 3 and 5 protein metabolism pathways were downregulated in SR-fed guts compared to paper and CS respectively. Two carbohydrate metabolism pathways were also downregulated in SR compared to paper and CS. Seven pathways belonging to signal transduction, energy metabolism, the excretory system, secondary metabolite production, and cofactor and vitamin metabolism were also down-regulated in SR-fed guts relative to paper and CS. Only 2 pathways were down-regulated in worker guts feeding on CS compared to paper.

**Table 2:** **Functional level differentials**

**Pathway**	**Class**	**CS/Paper**	**SR/Paper**	**SR/CS**
***Protein metabolism***				
Cysteine and methionine metabolism			0.46	
D-Glutamine and D-glutamate metabolism				0.10
Glycine, serine and threonine metabolism				0.70
Phenylalanine metabolism			0.11	0.24
Propanoate metabolism			0.39	0.63
Valine, leucine and isoleucine biosynthesis				0.46
***Carbohydrate metabolism***				
Fructose and mannose metabolism				0.63
Pyruvate metabolism			0.51	
***Others***				
Hematopoietic cell lineage	Immune system			**10.74**
HIF-1 signaling pathway	Signal Transduction			0.67
Legionellosis	Disease		0.48	0.67
Methane metabolism	Energy metabolism		0.44	0.61
Pathogenic Escherichia coli infection	Disease			0.60
Proximal tubule bicarbonate reclamation	Excretory System		0.42	0.59
Vasopressin-regulated water reabsorption	Excretory System			0.56
Streptomycin biosynthesis	Secondary metabolite production		0.50	
Thiamine metabolism	Cofactor and Vitamin metabolism		0.19	
Two-component system	Signal Transduction		0.35	
Meiosis - yeast	Cell Cycle	0.53		
Rheumatoid arthritis	Disease	0.56	0.49	

Differential pathways (edgeR, FDR p < 0.05) in the guts of *Reticulitermes flavipes* workers feeding on paper, Corn Stover (CS) or Soybean Residue (SR). Values greater or less than 1 are higher in the numerator or denominator of each respective comparison.

**Figure 4:**
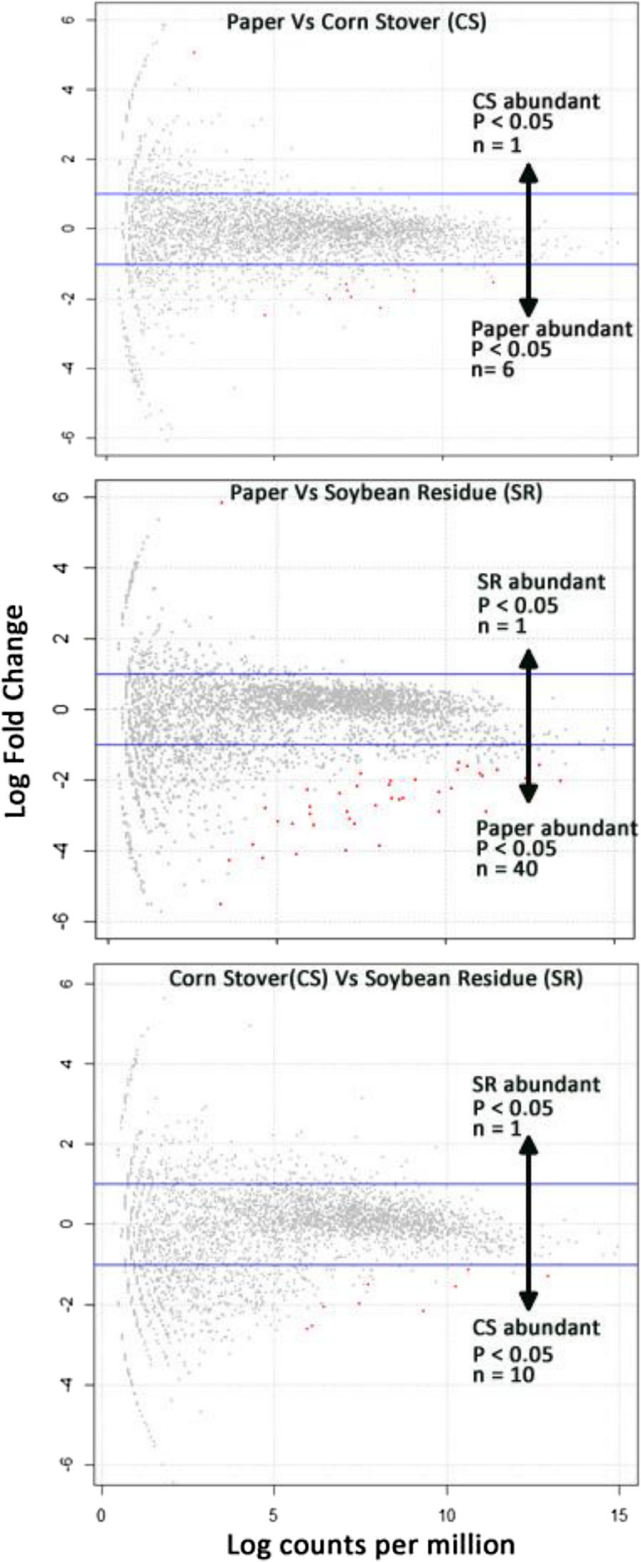
Pairwise comparions of gene expression among Paper, Corn Stover and Soybean feeding treatments. Plots show the expression profiles for non-significant (gray) and significant differentially expressed genes (Red) among the three pairwise comparisons. The x-axes represent the log_10_ read counts per million and the y-axes log_2_ fold change, for each KO term.

Forty-one unique KO terms transcripts were differentially expressed (edgeR, FDR < 0.05) in total among the three treatments (Figure 4, Additional file 1: Table S6A, B, C). Six and forty KO terms were downregulated in worker guts feeding on CS and SR respectively relative to paper. Ten KO terms were downregulated in SR compared to CS. Among the downregulated genes in the CS relative to paper treatment, the majority belonged to cell growth and death processes. By contrast, the majority of downregulated genes in the SR treatment had protein metabolism (6), carbohydrate metabolism (5) and energy metabolism (7) functions. Only a single gene was upregulated in each of the three pairwise comparisons. The *5-carboxymethyl-2-hydroxymuconate isomerase* gene was upregulated 5-fold in the CS treatment as compared to paper. A signaling gene, *MAPK41* (mitogen-activated protein kinase 1) was upregulated 5-fold in the SR treatment as compared to paper. Finally, *Condensin complex subunit* was up-regulated 10-fold in the SR treatment as compared to CS. Pairwise comparison of guts of SR and CS fed termites showed downregulation of 11 KO term in SR-fed guts relative to CS; or conversely, these 11 KO terms are expressed at higher levels in CS-fed compared to SR-fed guts. RT-qPCR was used to independently validate RNA-seq findings for the small number of passing candidate genes noted above, as well as non-passing potential lignocellulase genes identified through previous work (Additional file 2: Figure S5A, B, C). Expression patterns of the validated KO term transcripts were similar between RT-qPCR and RNA-seq; however there was no statistical correlation between fold changes of the two techniques. Alternatively, comparison of fold changes of RT-qPCR of the Illumina sequenced and independent biological replicates showed significant correlation (Additional file 2: Figure S6). Thus, collectively, these results provide evidence to conclude that RNA-seq results are valid.

## Discussion

Here we compared gut metatranscriptomes of *R. flavipes* workers feeding on different second generation feedstocks. Our goal was to identify the lignocellulase genes responding to these diets for their potential use in the pretreatment, and potentially saccharification processes of biofuel production. While our results suggest that CS and SR diets have little impact on termite digestome composition, our findings also suggest that the necessary lignocellulases required for digestion are constitutively expressed. However, we also found that the microbial gut community profiles of termites fed CS and SR diets shifted relative to those fed a paper diet. The CS metatranscriptome was functionally similar to that of the paper diet; whereas the SR metatranscriptome differed from the paper diet. It is notable that SR-fed termites were affected at the microbial community, pathway and functional levels. Protein and carbohydrate pathways were down-regulated in termites fed on SR; whereas, CS-fed termites exhibited minimal effects at the pathway and gene levels.

Interestingly, in the seven-day feeding bioassay, *R. flavipes* workers consumed more CS and SR than pure-cellulose paper (Figure 1A). Relative to paper, CS and SR have lesser carbohydrate and higher lignin content which might have resulted in higher consumption by termites to sustain themselves on these diets. The metatranscriptome profiles of both CS and SR-fed termites differed at the symbiont community level. A significant decrease in protist abundance in SR-fed termites relative to paper and CS-fed termites might be the effect of secondary compounds in SR. Isoflavones are the major secondary chemicals in SR, and in CS it is DIMBOA (hydroxamic acid, 2,4-dihydroxy-7-methoxy-1,4-benzoxazin-3-one). Several secondary plant metabolites are known to possess antimicrobial and anti-protozoal properties [34]. A study in ruminants has similarly shown a decrease in protozoan abundance upon feeding on certain plant materials [35]. A single study with the lower termite, *Kalotermes flavicollis*, showed a decrease in flagellate protists upon feeding on plant based essential oils [36]. However, there is no direct evidence suggesting antiprotozoal activity of soybean and corn extracts. Among the bacterial sequences obtained in this study there were few groups of bacteria that were significantly affected by the tested feedstocks. The effects of diet on the bacterial gut community cannot be reliably concluded as relatively few bacterial sequences with a potential polyA tail might have been sequenced. However, earlier findings by Boucias et al. [37] showed that feeding different lignocellulose diets for the same amount of time as the present study had minimal effects on the termite gut bacterial symbiont community [37].

At the pathway level (Table 2), the downregulation of protein and carbohydrate metabolism pathways in SR-fed termites might have been related to decreases in protist abundance. Several protists and their prokaryotic symbionts are well known to participate in protein and carbohydrate metabolism in termites [11,38]. A decline in the number of protists might have affected protein and carbohydrate metabolism in termites fed the SR diet. Pathway-level expression patterns were further supported by gene or function-level expression patterns. Genes participating in protein, carbohydrate and energy metabolism were downregulated in SR-fed termites relative to paper. Protein and carbohydrate metabolism pathways along with the genes participating in these pathways were more highly expressed in CS-fed termites relative to SR-fed termites. This further suggests that CS has less of a deleterious effect on the digestion mechanisms of *R. flavipes*.

There was one up-regulated gene in each treatment; *5-carboxymethyl-2-hydroxymuconate isomerase* was up-regulated in CS-fed termites, which acts on ring-opened metabolites of lignin phenolics [39] and participates in phenyl acetate catabolism for carbon in *E. coli* [40,41]. Upregulation of this gene either indicates the requirement of carbon by gut bacteria or a mechanism to cope with excess phenolic substrates encountered in the gut. *MAPK41,* upregulated in SR-fed termites relative to paper-fed, belongs to the superfamily of mitogen-activated protein kinases which are vital players in signal transduction. *MAPK41* along with other proteins regulate several cellular activities including transcription, cell division, metabolism, movement and apoptosis [42]. *Condensin complex subunit* was also more highly expressed in SR-fed termites compared to CS-fed termites. *Condensin complex subunit* is important for the organization of chromosomes during cell division and is ubiquitously distributed in archaea, bacteria and eukaryotes [43]. The higher expression of *MAPK41* and *Condensin complex subunit* is consistent with the possibility that there is a high turnover of cells in SR-fed termites.

Among the KO terms that had higher transcript levels in CS-fed relative to SR-fed termite guts, ~50% are terms related to amino acid metabolism and malate dehydrogenase isoforms. Higher levels of amino acid metabolism terms in CS-fed termites relative to SR-fed further supports the detrimental effects of SR compared to CS on termites and symbionts. Malate dehydrogenases are ubiquitously distributed and participate in a variety of metabolic pathways [44]. In prokaryotes, malate dehydrogenase plays a role in carbon fixation [44]. Higher expression of malate dehydrogenases in CS-fed termites compared to SR-fed suggests higher metabolism in termites or their symbionts with CS feeding.

Expression patterns similar to RNA-seq were observed for the tested KO term transcripts using RT-qPCR. However, EdgeR predicted differentials were not statistically significant in RT-qPCR, which is likely a result of differences in the normalization for the two methods. Further, the lack of correlation observed is due to differences in the magnitude of fold changes between the two techniques. However, a significant correlation was observed when using RT-qPCR to compare Illumina sequenced samples with independent biological samples (Additional file 2: Figure S6), which along with other results, provides reasonable validation of RNA-Seq results.

## Conclusion

In conclusion, this study provides a novel glimpse, using termites as a model system, into possible mechanisms of lignocellulosic feedstock digestion. The sequences obtained from this study also tremendously enrich existing *R. flavipes* sequence databases [16,23,45]. Our findings here were unexpected in that they did not support our initial hypothesis (i.e., that genes encoding stereotypical digestive and other biomass-active enzymes would be induced by feeding termites agricultural feedstocks). The metatranscriptomic profiles of CS and SR-fed termites were more similar than expected to those of paper-fed termites in terms of lignocellulase gene expression. Also, although SR effects were more severe, both feedstocks had negative impacts on protist abundance, as well as probable functions associated with protists. However, the CS and SR diets had no effect on the expression levels of stereotypical lignocellulases which were constitutively expressed. For example, a host endoglucanase identified through previous work, *Cell-1* [46], was constitutively expressed at high levels irrespective of the feedstock fed to termites. Prior *in vitro* analyses demonstrated the central role of Cell-1 in efficiently releasing glucose from wood substrates independent of symbiont action [16]. Also, termites are wood-feeding specialists and the genes that are used for wood digestion might be genetically determined to express constitutively and may therefore be non-responsive to CS and SR regulation. Thus, we hypothesize that constitutively expressed lignocellulases (Cell-1, aldo-keto reductases, laccases, catalases, etc. [16]) may serve as biocatalysts for both pretreatment and saccharification of CS and SR feedstocks. This hypothesis is being further tested at the translational level using proteomics, along with other integrative approaches, and will be the focus of a forthcoming report.

## Additional files

Additional file 1: Table S1. Primers used for RT-qPCR validation of RNA seq data. **Table S2.** Compositional analysis of paper, corn stover and soybean residue. **Table S3.** Illumina Sequencing summary of Paper, Corn Stover (CS) and Soybean Residue (SR) libraries. **Table S4A.** Annotation Summary of the treatment libraries – Paper, Corn Stover (CS) and Soybean Residue (SR). **Table S4B.** Summary of MG-RAST analysis. **Table S5.** ANOVA test carried out bacterial abundance in termite gut feeding on paper (P), corn (CS), and soybean residue (SR). **Table S6A.** Differentially expressed (edgeR, FDR < 0.05) KO terms in the guts of *Reticulitermes flavipes* workers feeding on paper and corn stover for 7 days. **Table S6B.** Differentially expressed (edgeR, FDR < 0.05) KO terms in the guts of *Reticulitermes flavipes* workers feeding on paper and soybean residue for 7 days. **Table S6C.** Differentially expressed (edgeR, FDR < 0.05) KO terms in the guts of *Reticulitermes flavipes* workers feeding on soybean residue and corn stover for 7 days. Additional file 2: Figure S1. Bioinformatic Analysis pipeline, **Figure S2.** Rarefaction curves representing the annotated species richness of the nine libraries including three replicates of paper, corn stover and soybean residue metatranscriptomes. **Figure S3.** Pie charts indicate the taxonomic distribution of the nine metatranscriptomes at the phylum level. **(A)** Paper-1 **(B)** Paper-2 **(C)** Paper-3 **(D)** Corn Stover-1 **(E)** Corn Stover-2 **(F)** Corn Stover-3 **(G)** Soybean Residue-1 **(H)** Soybean Residue-2 **(I)** Soybean Residue-3. **Figure S4.** Abundance of different bacterial taxa sequenced in the guts of worker termites (*Reticulitermes flavipes*)*,* fed on diets of paper, Corn Stover or Soybean Residue for 7 days, as determined from metatranscriptome sequence data. Statistical significance between the groups was tested by ANOSIM. **Figure S5.** RT-qPCR validations of RNA seq data. Correlation between RNA-Seq and RT-qPCR was calculated by Spearman correlation. Genes validated include: Endoglucanase (*Cell-1*), β-glucoasidase (*b-Glc*), Aldoketoreductase (*AKR*), Catalase (*CAT*), Malate Dehydrogenases (*MDH1*, *MDH2*, *MDH3*), Serine Acetyl Transferase (*Ser Actrans*), Satrch Synthase (*Starch Syn*). **Figure S6.** Comparison of average fold change of RT-qPCR of Illumina sequenced samples (Rep1) and experimental replicate (Rep2) across all the pairwise comparisons per gene. Genes validated include: Endoglucanase (*Cell-1*), β-glucoasidase (*b-Glc*), Aldoketoreductase (*AKR*), Catalase (*CAT*), Malate Dehydrogenases (*MDH1*, *MDH2*, *MDH3*), Serine Acetyl Transferase (*Ser Actrans*), Satrch Synthase (*Starch Syn*).
