# Supplementary material for: Metatranscriptomic profiles of Eastern subterranean termites, Reticulitermes flavipes (Kollar) fed on second generation feedstocks
Source: BMC Genomics. 2015 Apr 22;16(1):332. doi: 10.1186/s12864-015-1502-8 (PMC4411656; doi:10.1186/s12864-015-1502-8)
Supplement: Additional file 1: Table S1. — Primers used for RT-qPCR validation of RNA seq data. Table S2. Compositional analysis of paper, corn stover and soybean residue. Table S3. Illumina Sequencing summary of Paper, Corn Stover (CS) and Soybean Residue (SR) libraries. Table S4A. Annotation Summary of the treatment libraries – Paper, Corn Stover (CS) and Soybean Residue (SR). Table S4B. Summary of MG-RAST analysis. Table S5. ANOVA test carried out bacterial abundance in termite gut feeding on paper (P), corn (CS), and soybean residue (SR). Table S6A. Differentially expressed (edgeR, FDR < 0.05) KO terms in the guts of Reticulitermes flavipes workers feeding on paper and corn stover for 7 days. Table S6B. Differentially expressed (edgeR, FDR < 0.05) KO terms in the guts of Reticulitermes flavipes workers feeding on paper and soybean residue for 7 days. Table S6C. Differentially expressed (edgeR, FDR < 0.05) KO terms in the guts of Reticulitermes flavipes workers feeding on soybean residue and corn stover for 7 days. [file 12864_2015_1502_MOESM1_ESM.docx]

Additional file 1

Table S1: Primers used for RT-qPCR validation of RNA seq data

| **Gene** | **Primer** | **Primer Sequence** | **T_m_** |
| --- | --- | --- | --- |
| Translation elongation factor-δ | TEFD-F | CAGCATATGCAGCCAAGAAA | 63.8 |
|  | TEFD-R | AATGGCACAAACTTGGAAGC | 63.9 |
| Endoglucanase | Cell-1F | TCACAAGCAAGCAGGCATAC | 60 |
|  | Cell-1R | ATGAGAGCAGAATTGGCAGC | 60 |
| β-glucosidase | b-Glc-F | TGCTTCTTCATGGCTCAGAGT | 60 |
|  | b-Glc-R | TGGTCTCCAGGTTGTGTATCC | 60 |
| Aldoketo reductase | AKR-F | TGCTAGTCGTTCAGCATCCA | 60 |
|  | AKR-R | GAATTAAGCAGGCCCAGACA | 60 |
| Catalase | CAT-F | ACTTTGCTGAGGTGGAGCAG | 60 |
|  | CAT-R | GACGGTGCGTGTCTGAGTAG | 60 |
| Malate Dehydrogenase isoform 1 | MDH1-F | AGTGACAGGAGCCCTCAAGA | 64.1 |
|  | MDH1-R | GCCTCGCTTCTGAACAGTTG | 65.1 |
| Malate Dehydrogenase isoform 1 | MDH2-F | CGAACCAGATCAACAACGTG | 64.2 |
|  | MDH2-R | GCACATCTCCGGGTTGATA | 63.6 |
| Malate Dehydrogenase isoform 1 | MDH3-F | GCGACTACGCCAACCATATAA | 63.6 |
|  | MDH3-R | TCTTCATTTCGTCGCTGATG | 64 |
| Serine acetyl-transferase | Ser actrans-F | GCAACACTTGGTGGTACAGG | 64 |
|  | Ser actrans-R | TCGCCAATTGTGATATTTCC | 64 |
| Starch Synthase | Starch Syn-F | TTTTCGCTAAAGCATCTTTGAA | 62.6 |
|  | Starch Syn-R | ATGGCAGGAACAAAACCAGT | 63.3 |

Table S2. Compositional analysis of paper, corn stover and soybean residue

| **Sample** | **Glucan %** | **Xylan %** | **Lignin (gm/ gm CW*)** |
| --- | --- | --- | --- |
| Paper^φ^ | 98% | 0 | 0 |
| Corn Stover | 45.17 ± 1.49 | 28.93 ± 2.05 | 7.13 ± 0.81 |
| Soybean Residue | 44.0 ± 0.24 | 33.54 ± 0.89 | 6.36 ± 1.00 |

* CW = Cell Wall

^φ^ As per the manufacturer description. Whatman no.1 filter paper (Maidstone, UK)

Table S3: Illumina Sequencing summary of Paper, Corn Stover (CS) and Soybean Residue (SR) libraries

|  | **Raw reads** | | | **Quality & adapter clipped reads** | | | | |
| --- | --- | --- | --- | --- | --- | --- | --- | --- |
| **Sample** | **Sequences** | **Bases** | **Maximum length** | **Sequences** | **Bases** | **Minimum length** | **Mean Length** | **Max Length** |
| Paper-1 | 95,332,382 | 9,628,570,582 | 101 | 94,342,732 | 9,376,614,773 | 30 | 99 | 101 |
| Paper-2 | 99,637,096 | 10,063,346,696 | 101 | 98,568,900 | 9,779,621,162 | 30 | 99 | 101 |
| Paper-3 | 81,580,454 | 8,239,625,854 | 101 | 80,738,080 | 8,003,689,935 | 30 | 99 | 101 |
| CS-1 | 106,832,106 | 10,790,042,706 | 101 | 105,567,434 | 10,471,491,183 | 30 | 99 | 101 |
| CS-2 | 92,773,542 | 9,370,127,742 | 101 | 91,818,696 | 9,123,505,967 | 30 | 99 | 101 |
| CS-3 | 91,738,656 | 9,265,604,256 | 101 | 90,837,166 | 9,011,296,241 | 30 | 99 | 101 |
| SR-1 | 94,709,900 | 9,565,699,900 | 101 | 93,693,748 | 9,309,286,665 | 30 | 99 | 101 |
| SR-2 | 91,463,604 | 9,237,824,004 | 101 | 90,578,870 | 8,999,993,087 | 30 | 99 | 101 |
| SR-3 | 87,110,536 | 8,798,164,136 | 101 | 85,591,988 | 8,496,497,572 | 30 | 99 | 101 |

Table S4A. Annotation Summary of the treatment libraries – Paper, Corn Stover (CS) and Soybean Residue (SR)

| ***Analysis*** |  | MetaCV |  |  |  |  | BLASTN | RAPSearch2 |  |  |
| --- | --- | --- | --- | --- | --- | --- | --- | --- | --- | --- |
| ***Reference Database*** |  | Termite & Protist |  | Bacteria |  | Termite & Protist |  | nr |  | Total |
| **Sample** | **Raw Reads** | **Annotated** | **%** | **Annotated** | **%** | **Annotated** | **%** | **Annotated** | **%** | **%** |
| Paper1 | 93,162,046 | 16,086,542 | 17.3 | 1,653,548 | 1.77 | 31,080,228 | 33.36 | 8,852,664 | 9.5 | 61.91 |
| Paper2 | 97,169,452 | 17,563,850 | 18.1 | 2,282,848 | 2.35 | 33,244,590 | 34.21 | 6,908,126 | 7.11 | 61.75 |
| Paper3 | 79,520,362 | 14,462,998 | 18.2 | 1,690,284 | 2.13 | 28,797,306 | 36.21 | 5,857,852 | 7.37 | 63.89 |
| CS1 | 104,040,528 | 18,310,032 | 17.6 | 2,286,854 | 2.2 | 42,026,924 | 40.39 | 5,963,514 | 5.73 | 65.92 |
| CS2 | 90,610,978 | 14,300,740 | 15.8 | 1,767,480 | 1.95 | 38,185,178 | 42.14 | 5,367,300 | 5.92 | 65.8 |
| CS3 | 89,546,204 | 16,213,764 | 18.1 | 1,935,620 | 2.16 | 32,938,534 | 36.78 | 5,901,982 | 6.59 | 63.64 |
| SR1 | 92,487,706 | 14,673,224 | 15.9 | 1,657,972 | 1.79 | 31,991,152 | 34.59 | 8,603,526 | 9.3 | 61.55 |
| SR2 | 89,424,374 | 14,296,646 | 16 | 1,835,126 | 2.05 | 38,393,746 | 42.93 | 5,143,624 | 5.75 | 66.73 |
| SR3 | 84,339,230 | 13,323,456 | 15.8 | 1,139,794 | 1.35 | 37,892,016 | 44.93 | 4,809,632 | 5.7 | 67.78 |
| Total | 820,300,880 | 139,231,252 | 17 | 16,249,526 | 1.98 | 314,549,674 | 38.35 | 57,408,220 | 7 | 64.3 |

Table S4B: Summary of MG-RAST analysis

| **Sample** | **Total QC passed reads** | **Total reads after deduplication** | **Predicted ORFs** | **Identified proteins** | **Functionally annotated** | **% of total reads placed in pathways** |
| --- | --- | --- | --- | --- | --- | --- |
| **Paper1** | 46,581,023 | 27,068,977 | 23,537,621 | 4,963,809 | 2,952,811 | 5.68 |
| **Paper2** | 48,584,726 | 27,937,480 | 23,963,756 | 3,922,991 | 2,206,607 | 4.24 |
| **Paper3** | 39,760,181 | 21,880,016 | 18,985,704 | 3,461,859 | 2,039,972 | 3.92 |
| **CS1** | 52,020,264 | 26,751,661 | 22,215,674 | 4,034,547 | 2,231,546 | 4.29 |
| **CS2** | 45,305,489 | 23,827,953 | 19,450,336 | 3,605,740 | 2,022,367 | 3.89 |
| **CS3** | 44,773,102 | 24,843,804 | 20,910,487 | 3,726,034 | 2,100,871 | 4.04 |
| **SR1** | 46,243,853 | 26,392,745 | 22,654,581 | 4,679,098 | 2,724,878 | 5.24 |
| **SR2** | 44,712,187 | 23,124,049 | 18,959,966 | 3,499,865 | 1,976,150 | 3.8 |
| **SR3** | 42,169,615 | 21,099,413 | 17,476,951 | 3,202,023 | 1,835,368 | 3.53 |

|  | | | | |
| --- | --- | --- | --- | --- |
|  | | | | |
|  | | | | |
| Phyla |  | P | CS | SR |
|  |  |  |  |  |
|  |  |  |  |  |
| Proteobacteria |  | **213661±2938 a** | **167093±10862 b** | **131768±12997 b** |
| Bacteroidetes |  | **230749±13141 a** | **180420±18686 ab** | **122022±14922 b** |
| Bacteria |  | 5136±480 | 3057±941 | 3094±458 |
| Actinobacteria* |  | 107565±12411 | 99984±7582 | 67377±12018 |
| Firmicutes |  | **404755±30584 a** | **311166±30212 ab** | **233794±25306 b** |
| Spirochaetes |  | 62971±4945 | 69229±3827 | 39745±10494 |
| Tenericutes |  | 510±113 | 134±57 | 430±93 |
| Elusimicrobia |  | 6915±3777 | 11862±3080 | 2212±1470 |
| Dictyoglomi |  | 38±8 | 48±3 | 42±6 |
| Synergistetes |  | 2314±633 | 1813±608 | 1385±478 |
| Chrysiogenetes |  | 1676±41 | 1376±151 | 1195±154 |
| Deferribacteres* |  | **4514±145 a** | **3736±217 ab** | **3151±206 b** |
| Deinococcus_Thermus |  | **3345±569 a** | **2302±218 ab** | **1580±219 b** |
| Chloroflexi |  | **14297±905 a** | **7610±1127 b** | **6980±515 b** |
| Cyanobacteria |  | **18286±204 a** | **12361±1640 ab** | **11147±2107 b** |
| Verrucomicrobia |  | **10645±2364 a** | **6809±1091 ab** | **2792±1661 b** |
| Chlorobi |  | **18143±2209 a** | **12544±2272 ab** | **6144±783 b** |
| Chlamydiae |  | **3746±212 a** | **3063±114 b** | **782±29 c** |
| Nitrospirae |  | 42±7 | 45±4 | 37±6 |
| Planctomycetes |  | **21826±3058 a** | **15283±1668 ab** | **11751±1451 b** |
| Acidobacteria |  | **14583±826 a** | **11413±1189 ab** | **8987±1219 b** |
| Aquificae |  | 751±404 | 395±392 | 282±280 |
| Fusobacteria |  | 7661±952 | 5227±1181 | 4321±1148 |
| Thermotogae |  | 5029±828 | 5143±853 | 3914±734 |
| Fibrobacteres |  | 19570±2322 | 24183±2241 | 15919±2880 |
| Lentisphaerae |  | **3451±781 a** | **1739±174 ab** | **899±503 b** |
| Total |  | **1182180±69407 a** | **958035±71274 ab** | **681749±82230 b** |
|  | | | | |
|  | | | | |
| Data represented in the table are mean abundance values (±SE; n=3). Different letters (a,b and ab) within the same row indicate significant differences among fed on different diets at P-value <0.05 based on Tukey's studentized range test.* indicates data transformation | | | | |
|  | | | | |

Table S5. ANOVA test carried out bacterial abundance in termite gut feeding on paper (P), corn (CS), and soybean residue (SR)

Table S6A: Differentially expressed (edgeR, FDR < 0.05) KO terms in the guts of *Reticulitermes flavipes* workers feeding on paper and corn stover for 7 days.

| **KO term*** | **logFC*** | **logCPM*** | **P Value** | **FDR** | **System Description** | **Pathway Description** | **EC number** | **Gene ID** |
| --- | --- | --- | --- | --- | --- | --- | --- | --- |
| K01826 | 5.043054 | 2.615066 | 4.18E-05 | 0.036748 | Amino acid metabolism | Tyrosine metabolism [PATH:ko00350] | EC:5.3.3.10 | hpaF, hpcD; 5-carboxymethyl-2-hydroxymuconate isomerase |
| K00640 | -2.28079 | 8.153586 | 1.44E-05 | 0.015805 | Amino acid metabolism | Cysteine and methionine metabolism [PATH:ko00270] | EC:2.3.1.30 | cysE; serine O-acetyltransferase |
| K02210 | -1.7802 | 7.160713 | 5.67E-05 | 0.04154 | Cell growth and death | Meiosis - yeast [PATH:ko04113] | EC:3.6.4.12 | MCM7, CDC47; DNA replication licensing factor MCM7 |
| K02541 | -1.94604 | 7.292782 | 1.41E-05 | 0.015805 | Cell growth and death | Meiosis - yeast [PATH:ko04113] | EC:3.6.4.12 | MCM3; DNA replication licensing factor MCM3 |
| K06678 | -9.84862 | 5.148408 | 6.51E-17 | 2.86E-13 | Cell growth and death | Cell cycle - yeast [PATH:ko04111] | none | YCG1, CAPG; condensin complex subunit 3 |
| K00029 | -1.54765 | 11.5168 | 6.99E-05 | 0.043951 | Energy metabolism | Carbon fixation in photosynthetic organisms [PATH:ko00710] | EC:1.1.1.40 | E1.1.1.40, maeB; malate dehydrogenase (oxaloacetate-decarboxylating)(NADP+) |
| K01870 | -1.76653 | 9.154387 | 1.92E-06 | 0.00422 | Translation | Aminoacyl-tRNA biosynthesis [PATH:ko00970] | EC:6.1.1.5 | IARS, ileS; isoleucyl-tRNA synthetase |

*KO term= KEGG orthology ID

*logFC = Log Fold Change

*logCPM= log Counts Per Million

Table S6B: Differentially expressed (edgeR, FDR < 0.05) KO terms in the guts of *Reticulitermes flavipes* workers feeding on paper and soybean residue for 7 days.

| **KO term** | **logFC** | **logCPM** | **P Value** | **FDR** | **System Description** | **Pathway Description** | **EC number** | **Gene ID** |
| --- | --- | --- | --- | --- | --- | --- | --- | --- |
| K04408 | 5.837745 | 3.366105 | 1.40E-05 | 0.003633 | Signal transduction | MAPK signaling pathway [PATH:ko04010] | EC:2.7.11.1 | MAP4K1, HPK1; mitogen-activated protein kinase kinase kinase kinase 1 |
| K10408 | -1.52258 | 10.40385 | 0.00016 | 0.022019 | Neurodegenerative diseases | Huntington's disease [PATH:ko05016] | none | DNAH; dynein heavy chain, axonemal |
| K01738 | -1.60396 | 12.78157 | 0.000421 | 0.045205 | Amino acid metabolism | Cysteine and methionine metabolism [PATH:ko00270] | EC:2.5.1.47 | cysK; cysteine synthase A |
| K01835 | -1.62133 | 10.6612 | 8.49E-05 | 0.014424 | Biosynthesis of other secondary metabolites | Streptomycin biosynthesis [PATH:ko00521] | EC:5.4.2.2 | pgm; phosphoglucomutase |
| K07937 | -1.718 | 10.35824 | 0.000344 | 0.041008 | Infectious diseases | Legionellosis [PATH:ko05134] | none | ARF1; ADP-ribosylation factor 1 |
| K00029 | -1.73281 | 11.54422 | 0.000345 | 0.041008 | Energy metabolism | Carbon fixation in photosynthetic organisms [PATH:ko00710] | EC:1.1.1.40 | E1.1.1.40, maeB; malate dehydrogenase (oxaloacetate-decarboxylating)(NADP+) |
| K00025 | -1.82297 | 11.01301 | 4.35E-05 | 0.009112 | Excretory system | Proximal tubule bicarbonate reclamation [PATH:ko04964] | EC:1.1.1.37 | MDH1; malate dehydrogenase |
| K09571 | -1.82891 | 7.489039 | 0.000385 | 0.043659 | Endocrine system | Estrogen signaling pathway [PATH:ko04915] | EC:5.2.1.8 | FKBP4_5; FK506-binding protein 4/5 |
| K01900 | -1.91781 | 11.08181 | 2.29E-05 | 0.005312 | Carbohydrate metabolism | Propanoate metabolism [PATH:ko00640] | EC:6.2.1.4 6.2.1.5 | LSC2; succinyl-CoA synthetase beta subunit |
| K01624 | -1.95919 | 12.36783 | 5.94E-05 | 0.011523 | Energy metabolism | Methane metabolism [PATH:ko00680] | EC:4.1.2.13 | FBA, fbaA; fructose-bisphosphate aldolase, class II |
| K05349 | -1.99225 | 9.114214 | 1.40E-05 | 0.003633 | Biosynthesis of other secondary metabolites | Phenylpropanoid biosynthesis [PATH:ko00940] | EC:3.2.1.21 | bglX; beta-glucosidase |
| K07860 | -2.0325 | 8.373874 | 6.02E-05 | 0.011523 | Cardiovascular diseases | Viral myocarditis [PATH:ko05416] | none | RAC2; Ras-related C3 botulinum toxin substrate 2 |
| K01596 | -2.05005 | 13.39832 | 1.06E-05 | 0.003189 | Excretory system | Proximal tubule bicarbonate reclamation [PATH:ko04964] | EC:4.1.1.32 | E4.1.1.32, pckA, PEPCK; phosphoenolpyruvate carboxykinase (GTP) |
| K00335 | -2.14662 | 8.335895 | 0.00016 | 0.022019 | Energy metabolism | Nitrogen metabolism [PATH:ko00910] | EC:1.6.5.3 | nuoF; NADH-quinone oxidoreductase subunit F |
| K10410 | -2.18004 | 7.399778 | 0.000328 | 0.041008 | Neurodegenerative diseases | Huntington's disease [PATH:ko05016] | none | DNALI; dynein light intermediate chain, axonemal |
| K01903 | -2.23331 | 10.16183 | 0.000105 | 0.015909 | Energy metabolism | Carbon fixation pathways in prokaryotes [PATH:ko00720] | EC:6.2.1.5 | sucC; succinyl-CoA synthetase beta subunit |
| K11143 | -2.27818 | 5.92296 | 8.07E-05 | 0.014424 | Neurodegenerative diseases | Huntington's disease [PATH:ko05016] | none | DNAI2; dynein intermediate chain 2, axonemal |
| K01067 | -2.36146 | 9.796043 | 1.46E-06 | 0.000805 | Carbohydrate metabolism | Pyruvate metabolism [PATH:ko00620] | EC:3.1.2.1 | E3.1.2.1, ACH1; acetyl-CoA hydrolase |
| K01733 | -2.37701 | 6.885773 | 8.89E-05 | 0.014487 | Metabolism of cofactors and vitamins | Vitamin B6 metabolism [PATH:ko00750] | EC:4.2.3.1 | thrC; threonine synthase |
| K00703 | -2.50917 | 8.400336 | 1.09E-05 | 0.003189 | Carbohydrate metabolism | Starch and sucrose metabolism [PATH:ko00500] | EC:2.4.1.21 | E2.4.1.21, glgA; starch synthase |
| K00088 | -2.51359 | 8.741127 | 9.43E-07 | 0.000805 | Xenobiotics biodegradation and metabolism | Drug metabolism - other enzymes [PATH:ko00983] | EC:1.1.1.205 | guaB; IMP dehydrogenase |
| K10046 | -2.54133 | 8.65123 | 5.28E-06 | 0.001934 | Carbohydrate metabolism | Amino sugar and nucleotide sugar metabolism [PATH:ko00520] | EC:5.1.3.18 5.1.3.- | GME; GDP-D-mannose 3', 5'-epimerase |
| K01769 | -2.7182 | 7.942923 | 2.53E-06 | 0.001236 | Nucleotide metabolism | Purine metabolism [PATH:ko00230] | EC:4.6.1.2 | E4.6.1.2; guanylate cyclase, other |
| K07407 | -2.76435 | 6.006505 | 0.000129 | 0.018875 | Glycan biosynthesis and metabolism | Glycosphingolipid biosynthesis - globo series [PATH:ko00603] | EC:3.2.1.22 | E3.2.1.22B, galA, rafA; alpha-galactosidase |
| K00532 | -2.80484 | 4.682847 | 0.000387 | 0.043659 | Energy metabolism | Methane metabolism [PATH:ko00680] | EC:1.12.7.2 | E1.12.7.2; ferredoxin hydrogenase |
| K00027 | -2.89529 | 11.22287 | 2.11E-09 | 4.63E-06 | Signal transduction | Two-component system [PATH:ko02020] | EC:1.1.1.38 | E1.1.1.38, sfcA, maeA; malate dehydrogenase (oxaloacetate-decarboxylating) |
| K00024 | -2.89578 | 9.808569 | 3.87E-06 | 0.001549 | Energy metabolism | Methane metabolism [PATH:ko00680] | EC:1.1.1.37 | mdh; malate dehydrogenase |
| K00527 | -2.8968 | 7.082065 | 0.000421 | 0.045205 | Nucleotide metabolism | Pyrimidine metabolism [PATH:ko00240] | EC:1.17.4.2 | nrdD; ribonucleoside-triphosphate reductase |
| K00926 | -2.96485 | 5.983325 | 2.10E-05 | 0.005124 | Amino acid metabolism | Arginine and proline metabolism [PATH:ko00330] | EC:2.7.2.2 | arcC; carbamate kinase |
| K01676 | -3.11534 | 7.175095 | 8.85E-06 | 0.002996 | Energy metabolism | Carbon fixation pathways in prokaryotes [PATH:ko00720] | EC:4.2.1.2 | E4.2.1.2A, fumA, fumB; fumarate hydratase, class I |
| K07194 | -3.19377 | 5.05372 | 8.53E-05 | 0.014424 | Endocrine system | Insulin signaling pathway [PATH:ko04910] | none | RHOQ, TC10; Ras homolog gene family, member Q |
| K01912 | -3.24024 | 7.31554 | 2.22E-07 | 0.000245 | Amino acid metabolism | Phenylalanine metabolism [PATH:ko00360] | EC:6.2.1.30 | paaK; phenylacetate-CoA ligase |
| K03150 | -3.24608 | 5.471436 | 2.74E-05 | 0.006019 | Metabolism of cofactors and vitamins | Thiamine metabolism [PATH:ko00730] | none | thiH; thiamine biosynthesis ThiH |
| K03816 | -3.27735 | 6.107117 | 0.000178 | 0.023728 | Nucleotide metabolism | Purine metabolism [PATH:ko00230] | EC:2.4.2.22 | xpt; xanthine phosphoribosyltransferase |
| K00054 | -3.84137 | 4.311134 | 9.97E-05 | 0.015664 | Metabolism of terpenoids and polyketides | Terpenoid backbone biosynthesis [PATH:ko00900] | EC:1.1.1.88 | mvaA; hydroxymethylglutaryl-CoA reductase |
| K00640 | -3.85554 | 8.040636 | 6.27E-10 | 2.76E-06 | Amino acid metabolism | Cysteine and methionine metabolism [PATH:ko00270] | EC:2.3.1.30 | cysE; serine O-acetyltransferase |
| K01790 | -4.01767 | 7.066348 | 1.18E-06 | 0.000805 | Biosynthesis of other secondary metabolites | Streptomycin biosynthesis [PATH:ko00521] | EC:5.1.3.13 | rfbC; dTDP-4-dehydrorhamnose 3,5-epimerase |
| K12339 | -4.109 | 5.578684 | 1.70E-07 | 0.000245 | Amino acid metabolism | Cysteine and methionine metabolism [PATH:ko00270] | EC:2.5.1.47 | cysM; cysteine synthase B |
| K00878 | -4.20708 | 4.581579 | 1.42E-06 | 0.000805 | Metabolism of cofactors and vitamins | Thiamine metabolism [PATH:ko00730] | EC:2.7.1.50 | thiM; hydroxyethylthiazole kinase |
| K01750 | -4.27929 | 3.605555 | 0.000204 | 0.026337 | Amino acid metabolism | Arginine and proline metabolism [PATH:ko00330] | EC:4.3.1.12 | E4.3.1.12, ocd; ornithine cyclodeaminase |
| K01193 | -5.5438 | 3.328594 | 3.73E-06 | 0.001549 | Carbohydrate metabolism | Starch and sucrose metabolism [PATH:ko00500] | EC:3.2.1.26 | E3.2.1.26, sacA; beta-fructofuranosidase |

*KO term= KEGG orthology ID

*logFC = Log Fold Change

*logCPM= log Counts Per Million

Table S6C: Differentially expressed (edgeR, FDR < 0.05) KO terms in the guts of *Reticulitermes flavipes* workers feeding on soybean residue and corn stover for 7 days.

| **KO term** | **logFC** | **logCPM** | **P Value** | **FDR** | **System Description** | **Pathway Description** | **EC number** | **Gene ID** |
| --- | --- | --- | --- | --- | --- | --- | --- | --- |
| K06678 | 9.780812 | 5.23585 | 9.35E-21 | 4.11E-17 | Cell growth and death | Cell cycle - yeast [PATH:ko04111] | none | YCG1, CAPG; condensin complex subunit 3 |
| K00025 | -1.13885 | 10.62823 | 3.40E-05 | 0.021374 | Excretory system | Proximal tubule bicarbonate reclamation [PATH:ko04964] | EC:1.1.1.37 | MDH1; malate dehydrogenase |
| K01596 | -1.30719 | 12.95028 | 1.32E-05 | 0.009655 | Excretory system | Proximal tubule bicarbonate reclamation [PATH:ko04964] | EC:4.1.1.32 | E4.1.1.32, pckA, PEPCK; phosphoenolpyruvate carboxykinase (GTP) |
| K00703 | -1.4981 | 7.724145 | 7.20E-05 | 0.035207 | Carbohydrate metabolism | Starch and sucrose metabolism [PATH:ko00500] | EC:2.4.1.21 | E2.4.1.21, glgA; starch synthase |
| K00027 | -1.57298 | 10.25398 | 3.92E-07 | 0.000646 | Signal transduction | Two-component system [PATH:ko02020] | EC:1.1.1.38 | E1.1.1.38, sfcA, maeA; malate dehydrogenase (oxaloacetate-decarboxylating) |
| K01769 | -1.99236 | 7.468415 | 4.40E-07 | 0.000646 | Nucleotide metabolism | Purine metabolism [PATH:ko00230] | EC:4.6.1.2 | E4.6.1.2; guanylate cyclase, other |
| K01912 | -2.0694 | 6.452049 | 1.02E-05 | 0.008973 | Amino acid metabolism | Phenylalanine metabolism [PATH:ko00360] | EC:6.2.1.30 | paaK; phenylacetate-CoA ligase |
| K00024 | -2.15596 | 9.31109 | 9.24E-06 | 0.008973 | Energy metabolism | Methane metabolism [PATH:ko00680] | EC:1.1.1.37 | mdh; malate dehydrogenase |
| K00259 | -2.54177 | 6.094156 | 5.92E-05 | 0.032546 | Metabolism of other amino acids | Taurine and hypotaurine metabolism [PATH:ko00430] | EC:1.4.1.1 | ald; alanine dehydrogenase |
| K01790 | -2.62337 | 5.967439 | 9.13E-05 | 0.037069 | Biosynthesis of other secondary metabolites | Streptomycin biosynthesis [PATH:ko00521] | EC:5.1.3.13 | rfbC; dTDP-4-dehydrorhamnose 3,5-epimerase |
| K00800 | -3.23633 | 3.701724 | 9.27E-05 | 0.037069 | Amino acid metabolism | Phenylalanine, tyrosine and tryptophan biosynthesis [PATH:ko00400] | EC:2.5.1.19 | aroA; 3-phosphoshikimate 1-carboxyvinyltransferase |

*KO term= KEGG orthology ID

*logFC = Log Fold Change

*logCPM= log Counts Per Million
