# Supplementary material for: Metatranscriptomic profiles of Eastern subterranean termites, Reticulitermes flavipes (Kollar) fed on second generation feedstocks
Source: BMC Genomics. 2015 Apr 22;16(1):332. doi: 10.1186/s12864-015-1502-8 (PMC4411656; doi:10.1186/s12864-015-1502-8)
Supplement: Additional file 2: Figure S1. — Bioinformatic Analysis pipeline, Figure S2. Rarefaction curves representing the annotated species richness of the nine libraries including three replicates of paper, corn stover and soybean residue metatranscriptomes. Figure S3. Pie charts indicate the taxonomic distribution of the nine metatranscriptomes at the phylum level. (A) Paper-1 (B) Paper-2 (C) Paper-3 (D) Corn Stover-1 (E) Corn Stover-2 (F) Corn Stover-3 (G) Soybean Residue-1 (H) Soybean Residue-2 (I) Soybean Residue-3. Figure S4. Abundance of different bacterial taxa sequenced in the guts of worker termites (Reticulitermes flavipes), fed on diets of paper, Corn Stover or Soybean Residue for 7 days, as determined from metatranscriptome sequence data. Statistical significance between the groups was tested by ANOSIM. Figure S5. RT-qPCR validations of RNA seq data. Correlation between RNA-Seq and RT-qPCR was calculated by Spearman correlation. Genes validated include: Endoglucanase (Cell-1), β-glucoasidase (b-Glc), Aldoketoreductase (AKR), Catalase (CAT), Malate Dehydrogenases (MDH1, MDH2, MDH3), Serine Acetyl Transferase (Ser Actrans), Satrch Synthase (Starch Syn). Figure S6. Comparison of average fold change of RT-qPCR of Illumina sequenced samples (Rep1) and experimental replicate (Rep2) across all the pairwise comparisons per gene. Genes validated include: Endoglucanase (Cell-1), β-glucoasidase (b-Glc), Aldoketoreductase (AKR), Catalase (CAT), Malate Dehydrogenases (MDH1, MDH2, MDH3), Serine Acetyl Transferase (Ser Actrans), Satrch Synthase (Starch Syn). [file 12864_2015_1502_MOESM2_ESM.docx]

Additional file 2

Fig S1: Bioinformatic Analysis pipeline

Figure S2: Rarefaction curves representing the annotated species richness of the nine libraries including three replicates of paper, corn stover and soybean residue metatranscriptomes

Figure S3: Pie charts indicate the taxonomic distribution of the nine metatranscriptomes at the phylum level. (A) Paper-1 (B) Paper-2 (C) Paper-3 (D) Corn Stover-1 (E) Corn Stover-2 (F) Corn Stover-3 (G) Soybean Residue-1 (H) Soybean Residue-2 (I) Soybean Residue-3

Fig S4. Abundance of different bacterial taxa sequenced in the guts of worker termites (*Reticulitermes flavipes*)*,* fed on diets of paper, Corn Stover or Soybean Residue for 7 days, as determined from metatranscriptome sequence data. Statistical significance between the groups was tested by ANOSIM.


Fig S5: RT-qPCR validations of RNA seq data. Correlation between RNA-Seq and RT-qPCR was calculated by Spearman correlation. Genes validated include: Endoglucanase (*Cell-1*), β-glucoasidase (*b-Glc*), Aldoketoreductase (*AKR*), Catalase (*CAT*), Malate Dehydrogenases (*MDH1*, *MDH2*, *MDH3*), Serine Acetyl Transferase (*Ser Actrans*), Satrch Synthase (*Starch Syn*)

Fig S6: Comparison of average fold change of RT-qPCR of Illumina sequenced samples (Rep1) and experimental replicate (Rep2) across all the pairwise comparisons per gene. Genes validated include: Endoglucanase (*Cell-1*), β-glucoasidase (*b-Glc*), Aldoketoreductase (*AKR*), Catalase (*CAT*), Malate Dehydrogenases (*MDH1*, *MDH2*, *MDH3*), Serine Acetyl Transferase (*Ser Actrans*), Satrch Synthase (*Starch Syn*)
